# Practical Manual on Diseases of Women and Uterine Therapeutics

**Published:** 1892-03

**Authors:** 


*Practical Manual on Diseases of Women and Uterine Therapeutics.* By H. MACNAUGHTON JONES, M.D., M.Ch. Fifth Edition. Pp. xl., 704. London: Baillière, Tindall and Cox. 1891.

The simple fact that this work has gone through five editions in seven years speaks for itself. The book can certainly be recommended as giving in a small compass a clear and comprehensive view of the practice and science of diseases of women.

